# Characterization of Growth Hormone Resistance in Experimental and Ulcerative Colitis

**DOI:** 10.3390/ijms18102046

**Published:** 2017-09-23

**Authors:** Christoffer Soendergaard, Peter Helding Kvist, Peter Thygesen, Mats Reslow, Ole Haagen Nielsen, John Joseph Kopchick, Thomas Lindebo Holm

**Affiliations:** 1 Novo Nordisk A/S, Haemophilia Research, Maaloev 2760, Denmark; c.sondergaard@gmail.com (C.S.); PHKV@novonordisk.com (P.H.K.); PTT@novonordisk.com (P.T.); MRSL@pilapharma.com (M.R.); 2 Department of Gastroenterology, Herlev Hospital, Herlev 2730, Denmark; Ole.Haagen.Nielsen@regionh.dk; 3 Pila Pharma AB, 20512 Malmö, Sweden; 4 Edison Biotechnology Institute & Department of Biomedical Sciences, HCOM, Ohio University, Athens, OH 45701, USA; kopchick@ohio.edu

**Keywords:** experimental colitis, GH resistance, GHR, IGF-1, inflammation, long acting human GH, ulcerative colitis

## Abstract

Growth hormone (GH) resistance may develop as a consequence of inflammation during conditions such as inflammatory bowel disease, encompassing ulcerative colitis (UC). However, the specific role of the GH–insulin growth factor (IGF)-1-axis and/or the functional consequences of GH resistance in this condition are unclear. In situ hybridization targeting the GH receptor (GHR) and relevant transcriptional analyses were performed in patients with UC and in IL-10 knock-out mice with piroxicam accelerated colitis (PAC). Using cultured primary epithelial cells, the effects of inflammation on the molecular mechanisms governing GH resistance was verified. Also, the therapeutic potential of GH on mucosal healing was tested in the PAC model. Inflammation induced intestinal GH resistance in UC and experimental colitis by down-regulating GHR expression and up-regulating suppressor of cytokine signalling (SOCS) proteins. These effects are driven by pro-inflammatory mediators (tumor necrosis factor (TNF)-α, interleukin (IL)-1β and IL-6) as confirmed using primary epithelial cells. Treatment of experimental colitis with GH increased IGF-1 and body weight of the mice, but had no effects on colonic inflammation or mucosal healing. The high transcriptional similarity between UC and experimental colitis accentuates the formation of intestinal GH resistance during inflammation. Inflammation-induced GH resistance not only impairs general growth but induces a state of local resistance, which potentially impairs the actions of GH on mucosal healing during colitis when using long-acting GH therapy.

## 1. Introduction

Resistance to the actions of growth hormone (GH) is classically focusing on hepatic GH resistance. This condition is characterized by normal or elevated levels of GH with corresponding decreased insulin-like growth factor-1 (IGF-1) levels resulting from an impaired hepatic response to GH. GH resistance is naturally occurring as a mean to regulate the anabolic actions of GH to limit energy expenditure. Malnutrition, anorexia nervosa [1,2], poorly controlled type I diabetes [3], and disorders in the liver (e.g., chronic liver disease [4] or nonalcoholic fatty liver disease [5]) are associated with GH resistance. Additionally, states of systemic inflammation such as rheumatoid arthritis [6] or inflammatory bowel diseases (IBD) [7] may also induce GH resistance. Patients with IBD may, depending on the disease severity and intestinal location, experience combined inflammation and malnutrition, which additively impacts GH responsiveness [7,8].

IBD is comprised by the two main entities of ulcerative colitis (UC) [9] and Crohn’s disease (CD) [10], both showing rising global incidence rates [11]. Intestinal inflammation in UC, the main focus of this study, is confined to the epithelial lining and the lamina propria of the colon [12]. 

Epithelial restitution, or “mucosal healing”, is today considered the major success criteria in the treatment of IBD [13,14]. When evaluating the impact of inflammation on the cellular responsiveness to GH in patients with IBD, the effect on the mucosal layer and consequently the potential impact on mucosal healing is of central importance. Accordingly, our study focuses on the lamina propria and epithelial lining (where the inflammatory process is located) and aims at evaluating the effects of inflammation on local GH resistance in patients with UC.

Mechanistically, GH responsiveness and the GH–IGF-1-axis are subject to regulation, both in terms of negative feedback on GH and IGF-1 secretion, but also in terms of GH receptor (GHR) and IGF-1 receptor (IGF1R) signalling. During inflammation, mediators may impact the GHR in two distinct ways to induce GH resistance [15]. First, in vitro studies have shown that the mediators tumor necrosis factor (TNF)-α and interleukin (IL)-1β both impair liver GHR expression, causing blunting of the GH response, an effect which is potentiated when the mediators are used in combination [16,17,18,19]. Second, factors such as IL-6 and lipopolysaccharide (LPS) can regulate GH-induced signal transduction by inducing the expression of suppressor of cytokine signalling (SOCS) proteins, especially SOCS1 and SOCS3 [17,18,19,20,21]. These are negative regulators of Janus kinase (JAK)/Signal transducer and activator of transcription (STAT) dependent growth factor- and cytokine receptors and are important for down-regulation of cognate ligand induced intracellular signalling [22]. Additionally, IL-6 might inhibit GH-induced promoter/enhancer activity, by reducing STAT5 DNA binding and consequently inhibiting the effects of GH [20]. 

Ample evidence suggests that inflammation directly impacts GH-induced signalling and hence induces GH resistance. The majority of previous studies of inflammation-induced resistance have used hepatic GHR expression and IGF-1 expression/secretion as the primary readout. Therefore, the direct effects of inflammation on GHR signalling in peripheral tissues, especially the inflamed intestine, are still largely unexplored, even though intestinal GHR RNA and protein expression has been documented in the intestine [23,24].

Animal studies have shown that STAT5 signalling (induced by GH) enhances intestinal barrier function and mucosal healing [25,26]. Additionally, it was shown that GH boosts epithelial proliferation and has a beneficial effect on colonic histopathology scores in spontaneous colitis in IL-10 knock out (k.o.) mice. Also, GH responsiveness was reduced during active inflammation—an effect that is alleviated by treatment with anti-TNF-α. This indicates that systemic inflammation negatively impacts GHR signalling leading to GH resistance both systemically and locally in the colon [27,28]. 

Despite the positive effects of GH observed in animal models, clinical studies in IBD have, however, not shown the same convincing data. In human studies, no improvement of disease activity scores was observed following GH treatment [29,30,31,32,33]. Nevertheless, one study revealed an effect of GH administration in CD patients, where improved Crohn’s disease activity index (CDAI) scores were found following 4 months of treatment compared to placebo controls [34]. 

In this study we investigated the colonic GHR gene expression in humans and mice to reveal how it is affected by intestinal inflammation. Additionally, circulating inflammatory mediators were measured in the patients, and their impact on GHR function was evaluated using primary murine enteroids. The study focuses on the intestinal mucosa and evaluates the therapeutic effects of murine GH and long-acting pegylated human GH (PEG-hGH) on disease progression and mucosal healing. Collectively, the study sheds light on the intestinal GH/GHR biology during intestinal inflammation.

## 2. Results

### 2.1. Growth Hormone Receptor (GHR) Expression Is Reduced in Patients with Active Ulcerative Colitis (UC)

**Figure 1:**
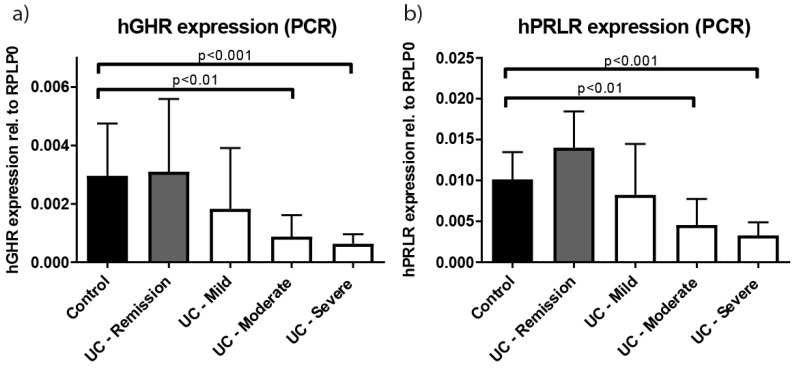
Gene expression levels (qPCR) of human growth hormone receptors (hGHR) (**a**) and human prolactin receptor (hPRLR) (**b**) in colonic biopsies from healthy controls (black), ulcerative colitis (UC) patients in remission (grey) or with active disease (white). A significant reduction in both RNA transcripts was observed for UC patients with moderate and severe disease compared to controls. Data presented as mean ± SD. Statistics: Kruskal–Wallis, Dunn’s correction, comparing all groups to control.

In a cohort of patients with UC and non-IBD controls (Table 1), the expression of GHR was evaluated in intestinal biopsies as determined by qPCR. RPLP0 (ribosomal protein, large, P0) was used as reference gene as it was not influenced by the presence of inflammation. Patients with moderate and severe UC showed significantly reduced GHR expression compared to both non-IBD controls and patients in remission (Figure 1a). As human GH also interacts with the prolactin receptor (PRLR), we analyzed its expression as well, which showed a similar significant reduction during moderate and severe inflammation compared to controls (Figure 1b).

**Figure 2:**
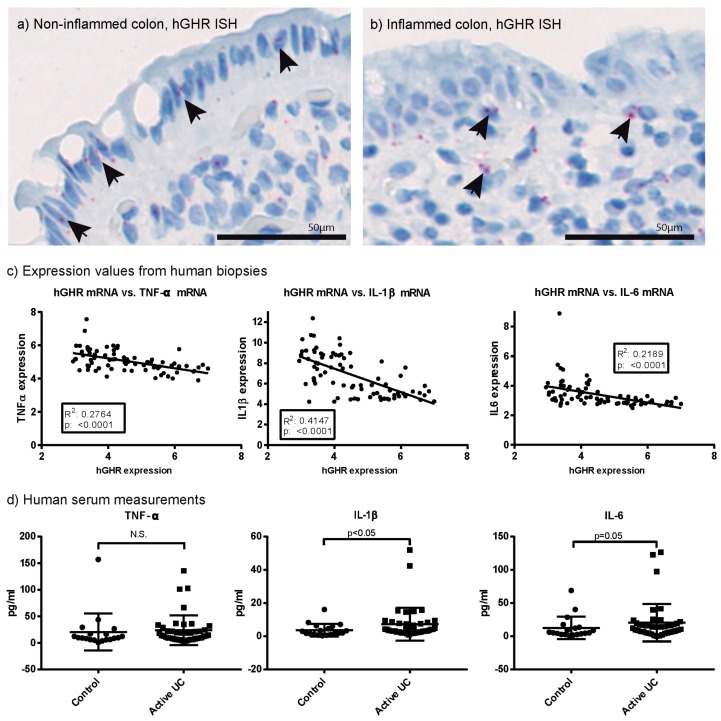
Expression of hGHR in human colon visualized by in situ hybridization in non-inflamed (**a**) and inflamed (**b**) UC specimens. Arrows indicate positively stained cells; (**c**) Correlation between the expression level of hGHR versus the expression of tumor necrosis factor (TNF)-α, interleukin (IL)-1β and IL-6 in colonic biopsies from the patient cohort (microarray data, log_2_-scale); (**d**) Serum measurements of TNF-α, IL-1β and IL-6 from non-inflammatory bowel disease (IBD) controls and patients with active UC. Data presented as mean ± SD. ISH: in situ hybridization. N.S.: not statistically significant. Statistics: simple linear regression and Mann–Whitney *U* test.

In situ hybridization (ISH) was conducted on biopsies from UC patients and controls to evaluate GHR expression in situ (Figure 2a,b). Validation of the applied probes is shown in Supplementary Materials Figure S1a. Evaluation of the stains focused on the epithelium and the lamina propria to which layers the inflammatory process is restriction in active UC. Generally, a low signal was obtained from the human biopsy material and, because of inconsistent orientation of the tissues, it was difficult to compare and quantitate staining intensity across samples. As a general trend, we observed that the GHR transcript was mostly expressed in the epithelial layer in non-inflamed samples, whereas less epithelial staining and more staining in cells in the lamina propria was observed during active disease (Figure 2a,b).

### 2.2. Systemic and Local Inflammatory Mediators Correlate Inversely with Colonic GHR Expression

Expression of the inflammatory mediators TNF-α, IL-1β and IL-6, were measured in the colonic biopsies from patients with UC and controls. All showed a statistically significant inverse correlation to the GHR expression levels (Figure 2c). Additionally, the GHR expression correlated inversely with both the endoscopic disease evaluation, histopathological evaluation and the global disease assessments using the Mayo score (Supplementary Materials Figure S2a–c). A higher coefficient of determination (R2) was observed between GHR expression and expression of the TNF-α and IL-1β (Figure 2c) compared to the correlation between GHR expression and the standard clinical Mayoscore (Supplementary Materials Figure S2a). This indicates that specific inflammatory pathways and the local inflammatory process have a more pronounced impact on colonic GHR expression than the overall disease severity. Systemically, we measured the short lived inflammatory mediators TNF-α, IL-1β and IL-6 (Figure 2d). In the patients with active UC, IL-1β was significantly elevated, IL-6 showed a borderline insignificant change and TNF-α was unchanged.

### 2.3. Characterisation of GHR Expression in Experimental Colitis

**Figure 3:**
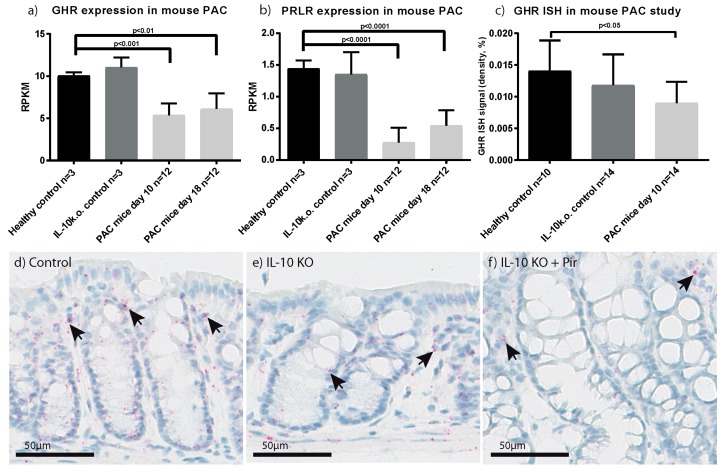
Colonic samples from the piroxicam accelerated colitis (PAC) model analyzed for expression of mouse growth hormone receptor (mGHR) (**a**) and mouse prolactin receptor (mPRLR) (**b**) by RNA seeq. Samples were obtained from healthy controls, IL-10 k.o. animals and after the indicated number of days following induction of disease using piroxicam; (**c**) Quantification of the in situ hybridization performed in the PAC model at day 10 (**d**–**f**). Arrows indicate positively stained cells. Data presented as mean ± SD. Statistics: Ordinary one way ANOVA with Holm Sidak’s correction comparing healthy control to all other groups.

We used IL-10 k.o. mice with piroxicam accelerated colitis (PAC), to evaluate the influence of active colonic inflammation on GHR expression in vivo (see experimental setup #1 in Section 4.6). The PAC model showed decreased GHR expression levels after both 10 and 18 days compared to healthy controls and IL-10 k.o. mice. No decrease was observed in the IL-10 k.o. background animals compared to healthy controls (Figure 3a). In the PAC model, an inverse correlation was observed between GHR expression and both TNF-α, IL-1β and SOCS3 as well as the histopathology score (Supplementary Materials Figure S3). Similarly, expression of PRLR (Figure 3b) revealed a profile mirroring GHR expression (Figure 3a). 

GHR expression in the PAC model was further validated by ISH staining (Figure 3c–f). The in situ staining was analysed using quantitative image analysis based on a previously validated method [35], which confirmed the observed decrease in GHR expression at day 10 compared to healthy controls (Figure 3c). In the murine colonic tissue, GHR expression was observed primarily in the epithelial lining of the intestine but also in single cells in the lamina propria in both healthy controls and during inflammation.

### 2.4. Inflammation Impacts Epithelial GHR Expression and Signalling

To evaluate the impact of inflammation on GHR expression in the intestinal epithelium, epithelial enteroid cultures from mice were established. We evaluated the direct effects of inflammation on both GHR expression and its function by assaying downstream STAT5 phosphorylation. Based on prior publications, we decided to evaluate the effects of TNF-α and IL-1β collectively and IL-6 separately, as these mediators impact GHR expression and signalling through separate mechanisms [15].

**Figure 4:**
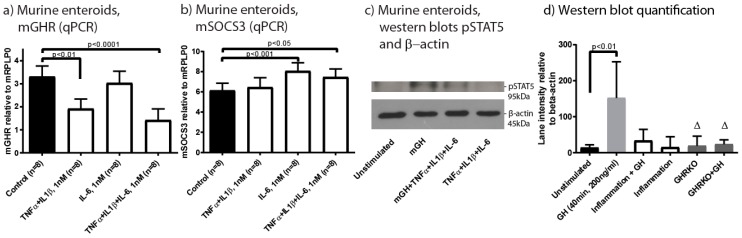
qPCR measuring mGHR (**a**) and mouse suppressor of cytokine signalling (mSOCS)3 (**b**) expression in murine enteroids. The cells were grown under standard conditions (black) or being stimulated with either TNF-α and IL-1β, with IL-6 or with all three mediators, 1 nM for 8 h, *n* = 8; (**c**) Western blotting of phosphorylated STAT5 and β-actin in murine enteroids to evaluate the effect of mGH alone and following pre-incubation with inflammatory mediators (TNF-α, IL-1β and IL-6) (1 nM of each in combination); (**d**) Quantification of western blots (*n* = 4). Intestinal organoids from gene disrupted mice (GHRKO) were also included. Δ only two samples—therefore not included in statistics. Data presented as mean ± SD. Statistics: Kruskal-Wallis, Dunn’s correction, comparing all groups to Control/Unstimulated.

We chose to culture murine small intestinal enteroids as their cellular differentiation is not hampered by exogenous Wnt3a addition through activation of the stem cell promoting Wnt pathway. Enteroid experiments were initiated by epidermal growth factor (EGF) starvation to limit basal STAT5 phosphorylation prior to inflammatory stimulation and expression analysis by PCR (Figure 4a,b). TNF-α and IL-1β (1 nM) induced a significant decrease in GHR expression, while IL-6 (1 nM) did not affect GHR expression (Figure 4a). A combination of the three mediators showed a synergistic repressive effect on the GHR expression (Figure 4a). IL-6 induced expression of SOCS3, both alone and in combination with TNF-α and IL-1β (Figure 4b), pointing to separate mechanisms being utilized by the different inflammatory mediators. STAT5 phosphorylation induced by mouse GH (mGH) in the enteroids was also repressed by an 8 h pre-incubation with 1 nM of each of the inflammatory mediators in combination as shown by western blotting (Figure 4c,d). Phosphorylation of STAT5 was induced through the GHR as enteroids derived from GHR k.o. mice did not respond to mGH (Figure 4d).

### 2.5. Gene Expression Signature Similarities between Human UC and Murine Experimental Colitis

To investigate the specific mechanisms governing the impact of inflammation on GHR functionality, gene expression signatures of relevant transcripts were compared between the UC patients, the murine PAC model and the murine enteroid system and presented in Table 2.

The results show a number of similarities in the cellular response to inflammation. Besides similar regulation of GHR and PRLR as described, all three systems show a similar inflammation-dependent increase in SOCS1 and SOCS3, expected to occur during inflammation (Table 2). The inflammatory mediators, TNF-α, IL-1β and IL-6 were up-regulated in UC and PAC at day 10. SOCS2 showed an inflammation-dependent decrease in UC and PAC at day 10, which is in contrast to SOCS1 and SOCS3. No changes in colonic IGF-1 expression were observed in any of the systems. IGF1R expression was decreased in both UC and PAC day 10, whereas IGF1R expression in murine enteroids was increased upon inflammation.

### 2.6. Therapeutic Treatment with High Dose Mouse GH (mGH) in Piroxicam Accelerated Colitis

Therapeutic treatment using high dose mGH in experimental colitis was initially evaluated in the PAC model (see experimental setup #2 in Section 4.6). A therapeutic study design was selected to prevent the effects that GH-treatment may induce on eating behaviour, size and metabolism of the animals, which bias the disease induction when using piroxicam containing chow.

As expected, high dose mGH (30 mg/kg) given twice daily by s.c. injection induced a significant weight gain (Supplementary Materials Figure S4a,b) and increased serum IGF-1 (Supplementary Materials Figure S4c) compared to vehicle- and anti-IL-12p40-treated animals. Thus, we confirm that mGH was biological active in vivo during active experimental colitis. No treatment effect on body weight following anti-IL-12p40 (monoclonal antibody) administration could be detected (Supplementary Materials Figure S4b). Treatment effects on the colon were also evaluated by histopathology score and colon weight:length ratio (Supplementary Materials Figure S4d,e). No treatment effect of mGH could be observed, whereas in anti-IL-12p40 treated mice a decreased disease severity was seen as previously described [36,37].

Disease induction in this study seemed to be mild with an average weight loss around 2% at the time of first treatment. Moreover, several mice in the vehicle treated group did not display evident signs of colitis at the time of euthanization.

### 2.7. Therapeutic Treatment with a Long Acting PEG-hGH in Piroxicam Accelerated Colitis

In a follow-up study, we modified the experimental design so that only mice with a weight loss of 7.5% were included in the treatment randomization. Additionally, high dose mGH was replaced by high dose long acting PEG-hGH in order to test chronic exposure of GH in the intestine to increase the direct GH-mediated effects on disease resolution (see experimental setup #3 in Section 4.6). Accordingly, we expected to increase the chances of observing an effect of PEG-hGH on mucosal healing. 

**Figure 5:**
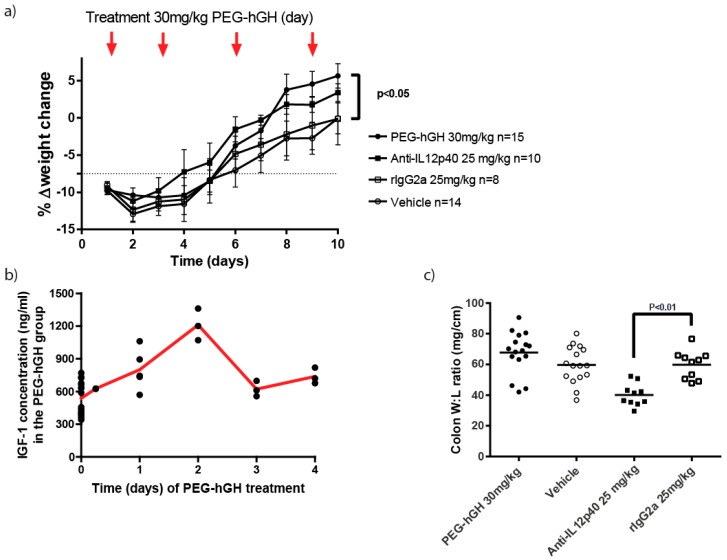

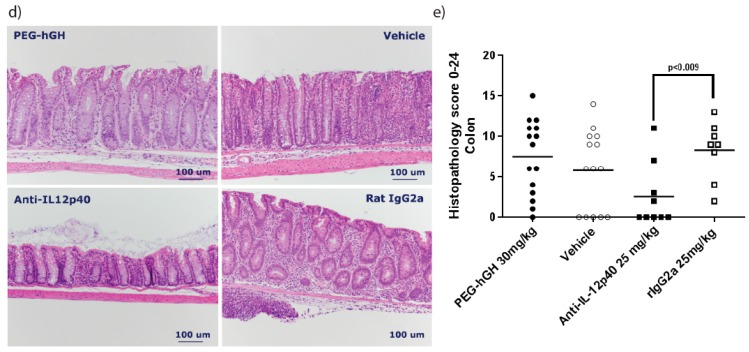
Therapeutic evaluation of the effects of pegylated-hGH (PEG-hGH) in the PAC model with established colitis. Arrows indicate dosing with PEG-hGH. (**a**) Weight change (% relative to pre-dosing with piroxicam) during the study with indications of dosing with PEG-hGH. The four treatment groups are indicated; (**b**) Plasma IGF-1 profile from the PEG-hGH treated group. Following PEG-hGH injection a sustained IGF-1 response is observed the following days; (**c**) Colonic weight:length ratio of the treated groups measured at day 10; (**d**) Representative colonic HE stains of the 4 treatment groups; (**e**) Histopathological scoring of colonic disease severity evaluated at day 10. Mean values are presented with SEM in (**a**). rIgG: rat IgG antibody.

Similar to the initial treatment study using mGH, therapeutic PEG-hGH treatment resulted in a significant body weight increase compared to vehicle treated mice, and an increase in IGF-1 concentration for 3 days compared to predose levels (Figure 5a,b). No significant effect of anti-IL-12p40 could be observed (Figure 5a). Treatment with PEG-hGH showed no effect on the colonic inflammation, i.e., no effect on colonic weight:length ratio or histopathology (Figure 5c–e). Thus, neither mGH nor PEG-hGH affected colonic inflammation in the PAC IL-10 k.o. mouse model.

## 3. Discussion

Inflammation is a known inducer of GH resistance, exemplified by growth retardation in up to one-third of pediatric cases of CD [38]. Development of growth retardation and GH resistance during inflammation likely serves a purpose of retaining energy during times of excessive catabolism and malnutrition. Accordingly, this is a result of a complex mechanism including both the effects of malnutrition and the impact of inflammatory mediators that affect parameters downstream of GH.

In the current study, we aimed at evaluating the physiological responses of inflammation on GH action in the intestine. Also, the potential impact of GH on the process of mucosal healing was evaluated. We focused on UC, as the inflammatory process in this condition is restricted to the epithelial lining and the lamina propria, which is represented in intestinal biopsies. Additionally, we compared the findings to animal models of experimental colitis in which we tested the therapeutic potential of GH.

Patients with active UC show a severity-dependent decrease in colonic GHR expression (Figure 1a and Supplementary Materials Figure S2a) which was also observed in our experimental PAC colitis model (Figure 3a). Besides a general inflammation-induced reduction in intestinal GHR expression, cell-type specific changes were also observed using ISH. Decreased epithelial expression and increased numbers of GHR positive cells in the lamina propria was observed in inflamed human intestines, which points to the induction of local epithelial GH resistance and infiltration of GHR positive immune cells into the lamina propria (Figure 2a). This is underlined by previous reports showing high GHR expression by immune cells, notably macrophages, neutrophils and B-cells [39,40,41]. Based on PCR analysis of human biopsies, the loss of GHR expression by epithelial and stromal cells is greater than the gain introduced by infiltrating immune cells during active UC. A similar pattern was observed in the PAC model where inflammation significantly reduced GHR expression (Figure 3c–f). 

The best studied inflammatory mediators involved in GH resistance formation are TNF-α, IL-1β and IL-6. Only IL-1β was up-regulated in serum from the UC patients (Figure 2d), whereas local expression of all three was increased in both active UC and in PAC mice (Table 2), which additionally inversely correlated with human GHR expression (Figure 2c). Combined with the consistent changes in gene expression patterns between UC patients and the PAC model, GH resistance appears to have been introduced (Table 2), where local TNF-α, IL-1β and IL-6 induce SOCS1 and SOCS3 and decrease GHR expression.

During inflammation SOCS proteins function as inhibitors of various cytokine receptors thus limiting hyper activation of inflammation-related cytokine-induced signalling through the JAK/STAT pathway. Consequently, GHR/JAK2/STAT5 signalling is collaterally targeted by the SOCSs as confirmed in vitro using the enteroid system (Figure 4c,d). A similar pattern with both up-regulation of SOCS1 and SOCS3 has been described among UC patients [42,43] and increased SOCS3 was reported in experimental colitis [27]. 

Change in colonic IGF-1 mRNA was not observed, whereas IGF1R expression was decreased in vivo (Table 2). The mechanism for this is unknown, but could be a consequence of either the inflammation per se or reduced IGF-1 levels resulting from induced hepatic GH resistance [44,45,46]. Chesnokova et al. [47] have reported enhanced intestinal GH protein levels in active UC based on immunohistochemical staining. By qPCR we detected no expression of GH1 in human and mice intestines, while GH2 was expressed based on array analyses in humans (Table 2). No changes in the expression was observed in non-inflamed versus inflamed samples neither in UC or PAC, indicating that local GH production is not influenced by intestinal inflammation.

Mucosal healing is the primary treatment goal in IBD and is defined as the reestablishment of the epithelial barrier. To evaluate the effects of inflammation and GH on the epithelial lining, we established primary epithelial cultures from mice [48]. Separate effects of TNF-α and IL-1β or IL-6, respectively, on regulation of GHR functions were observed. The effects on GHR and SOCS3 expression, already observed in vivo, combined with a negative impact on STAT5 phosphorylation indicates the induction of GH resistance in the cultured cells.

Previous studies have reported proliferative and anti-apoptotic effects of GH on murine intestinal epithelium and in human duodenal explant cultures [27,49,50]. In intestinal epithelium, activation of STAT5 has additionally been shown to be important for maintaining stem cell renewal and for crypt regeneration during intestinal inflammation, which further underlines the potential roles of GHR activation during mucosal healing [51]. 

To evaluate the therapeutic potential of GH, we used the PAC model. The model generally displays a more synchronized disease development compared to IL-10 k.o. mice on a C57BL/6J background, and shares many features with CD and UC as previously described [36,37]. The treatment design was inspired by previous reports showing that GH overexpressing mice, had increased survival, induction of remission and mucosal healing in dextran sulfate sodium (DSS) treated animals [52]. Moreover, Denson et al., and Williams et al., have previously evaluated the effects of GH on intestinal inflammation in pre-clinical models of colitis [27,28,52,53]. 

Contrary to previous studies, our model design was based on an accelerated disease using piroxicam. GH treatment was initiated after disease induction to evaluate its effects on established inflammation in a therapeutic model [36,37]. Additionally, we treated the mice with higher doses of GH compared to previous studies. The intention was to be able to override the induced GH resistance in the animals to observe an effect of GH on intestinal inflammation.

Twice daily treatment with high dose mGH significantly increased body weight and plasma IGF-1 but did not affect histopathological disease evaluation or colon weight:length ratio (Supplementary Materials Figure S4). In the initial study inconsistent disease penetration was observed, with low initial weight loss, where several animals did not show signs of colitis (Supplementary Materials Figure S4d,e). Treatment of the animals with anti-IL-12p40 did ameliorate the disease (Supplementary Materials Figure S4d,e). Accordingly, the model was adjusted to ensure homogenous disease severity and group assignment was initiated when the animals reached a 7.5% weight loss (Figure 5). Additionally, long acting PEG-hGH was used to increase the exposure to GH persistently during the treatment and to reduce the number of injections. PEG-hGH induced weight gain and increase in IGF-1 concentration in the treated animals, but histologically, the animals did not benefit from PEG-hGH treatment and the colonic weight:length ratio was not improved either.

In our study GH treatment (mGH and PEG-hGH) showed no effects on intestinal healing although the animals were exposed to high dose GH, indicated by increased body weight and IGF-1 concentration. It should be noted that the observed body weight increase likely is a combination of “growth” of the animals but also an effect of GH on sodium and water retention, a known effect of GH [54]. Similar to previous studies, we confirmed that inflammation induces GH resistance. However, in contrast to previous studies where prophylactic treatment or GH overexpression did ameliorate inflammation [27,28,52], we used a therapeutic setup, where GH resistance was established prior to treatment. It is likely that GH administration alone is not sufficient to circumvent established GH resistance resulting in limited GH actions at the site of active inflammation.

Whereas a number of studies have shown an effect of GH on experimental colitis, only one study has reported an effect of GH on CD colitis. In that study, Slonim et al. [34] observed reduced disease scores as well as a decreased need for concomitant medication following 4 months of GH supplementation as compared to placebo.

Malnutrition is an important factor in the formation of GH resistance, especially during intestinal inflammation, where nutritional uptake is likely impaired. The nutritional state was not possible to evaluate in the current study. However, prolonged disease duration and severity are risk factors for development of malnutrition in IBD in general [55,56]. Among UC patients included in our study, a decline in appetite and general well-being was observed, especially among patients with moderate and severe disease stages. Some degree of malnutrition must be expected in some of these patients. Likewise, severely diseased animals also consume less food although the study setup did not focus on this aspect. 

In conclusion, no effects of GH on intestinal mucosal healing were observed during inflammation, even though an effect on weight gain was observed showing a systemic biological effect of GH. Therefore, we believe that ongoing intestinal inflammation induces both global and local intestinal GH resistance.

## 4. Materials and Methods

### 4.1. Bioethics

All murine experiments were carried out in accordance with the legislation of the European Communities Council Directive 2010/63/EU for the protection of animals used for experimental purposes and approved by the Danish Animal Experiments Inspectorate, Ministry of Food, Agriculture and Fisheries, Denmark, as well as the internal Ethical Review Committee at Novo Nordisk A/S. Animal experiment permit numbers are: 2008/561-1510 and 2013-15-2934-00816/BES. Mice were sacrificed by cervical dislocation if their weight loss exceeded 20% within the study or if they showed signs of decreased well-being or a morbid appearance.

### 4.2. Patient Material and Ethical Considerations

The enrolled individuals were all contacted in relation to an already scheduled endoscopic examination at Herlev University Hospital, University of Copenhagen, Denmark. Signed informed consent was obtained from each individual before inclusion. Healthy volunteers consisted of patients with irritable bowel syndrome [57] or patients undergoing a scheduled control visit following a previous polypectomy. Demographics and clinical characteristics are described in details in Table 1 and have also been described previously [58,59]. Multiple intestinal biopsies were sampled from each patient within a 5 cm radius. The sampling was done from the sigmoid colon from the most inflamed area. The Scientific Ethics Committee of the Copenhagen Capital Region approved this study (H-2-2012-026 and H-15009463). The procedures applied were in accordance with the Declaration of Helsinki of the World Medical Association.

### 4.3. Clinical Disease Scoring of IBD Patients

Patients were evaluated during the visit at the endoscopy unit based on the Mayo score [60]. Three neighboring biopsies from each patient were evaluated by the staff pathologist and scored according to the histopathological Geboes-system [61]. This scoring system was used as a cumulative sum-score of the individual grades, thus ranging from 0 to 22. Based on the Mayo score (scores 0–12), the patients were graded as being in remission (0–2, no subscore > 1) or having mild (3–5), moderate (6–10) or severe (11–12) disease.

### 4.4. Animal Studies and Procedures

B6.129P2-*Il10138*^tm1Cgn^/J (IL-10 k.o.) mice and C57BL/6J wild type (WT) mice were obtained from Charles River Laboratories (Sulzfeld, Germany) in accordance with a license agreement with MCG (MCG-stiftung Hercogstrasse 64, 80803 Murnich, Germany). 8–12 week old mice, all of them female, were used in the studies due to the impracticalities of randomizing and housing male mice. The mice were housed at Novo Nordisk A/S, Maaloev, Denmark, under barrier protected conditions as described previously [37]. The animals were not exposed to agents listed in the FELASA (Federation of Laboratory Animal Science Associations) guidelines [62]. They were housed under a 12-h light/dark cycle with 10–12 mice per cage. 90 percentage of the cage bedding was changed weekly, and 10 percentage of the cage bedding were transferred between cages to ensure a homogenous microbial environment. The clinical status of the mice was evaluated three times weekly by visual inspection, percentage weight change and fecal consistency.

### 4.5. Induction of Piroxicam Accelerated Colitis (PAC)

**Figure 6:**
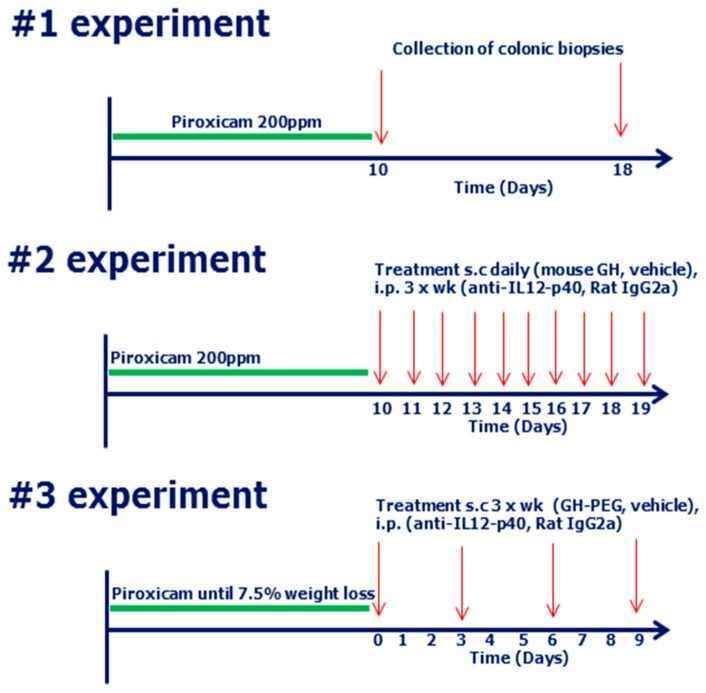
Graphical presentation of the in vivo protocols used in the study. Experiment 1 was used for gene expression profiling with arrows indicating euthanization of the animals (see Figure 4 and Table 2). Experiment 2 and 3 are dosing studies with mGH (see Supplementary Materials Figure S4) and PEG-hGH (Figure 5), respectively, with arrows indicating dosing with the described compounds.

For induction of the PAC IL-10 k.o. model, IL-10 k.o. mice had unrestricted access to piroxicam (Sigma Aldrich, Broendby, Denmark) containing 200 ppm homogenized in 1324 Altromin diet (Altromin, Lage, Germany) from day 0 of study initiation until day 10 (first and second experiment, see Figure 6), and from day 0 until they reached 7.5% weight loss (third experiment). Subsequently, mice were switched to normal Altromin 1324 chow as previously described [36,37]. 

In the second experiment, PAC mice were treated with mouse growth hormone (mGH) (30 mg/kg) or vehicle twice daily. The control treatment groups received anti-IL-12p40 (25 mg/kg) or rat IgG2a (25 mg/kg) three times a week after termination of piroxicam treatment.

In the third experiment, PAC mice were treated with long-acting PEG-hGH (30 mg/kg), vehicle, anti-IL-12p40 (25 mg/kg) or rat IgG2a (25 mg/kg) day 1, 3, 6 and 9 after termination of piroxicam treatment. Anti-IL-12p40 and rat IgG2a were purchased from BioXcell (West Lebanon, NH, USA) and mGH and PEG-hGH were produced in-house by Novo Nordisk (Bagsværd, Denmark).

### 4.6. Study Design

A graphical presentation of the three applied study designs is shown in Figure 6.

### 4.7. Monitoring of Disease in Mice

Body weight was monitored three times weekly, and mice were sacrificed at weight loss >20%. Fecal consistency was evaluated before the start of treatment and subsequently 3 times a week using a semi-quantitative score (normal stool = 0; slightly soft = 1; soft but formed = 2; not formed = 3; liquid stools or no feces in colon at sacrifice = 4). Disease activity index (DAI) score was calculated as previously described [38,39].

### 4.8. IGF-1 Measurements in Mice

Blood samples were drawn at predesignated time points pre- and post-dosing using a sparse sampling schedule. At each time point blood samples were drawn from 3 mice, whereas each mouse was bled 1–2 times during each study. Blood samples were drawn from the orbital vein plexus of animals anaesthetized by Isofluran/O2/N2O. At each sampling time 150 μL blood was drawn in an Eppendorff tube containing 8 mM EDTA. Plasma was collected after centrifugation at 1500× *g* at 4 °C for 10 min. A plasma sample of 25 μL was used to determine the IGF-1 concentrations by a commercial ELISA assay (OCTEIA IGF-I, IDS Ltd., Boldon, UK). The assay was a sandwich ELISA using a highly IGF-I specific polyclonal antibody as catcher, and a horseradish peroxidase labelled high affinity monoclonal antibody as detector. The limit of detection for IGF-I was 63 ng/mL.

### 4.9. Post Mortem Analyses on Mouse Tissue

As described previously [63] mice were sacrificed by cervical dislocation. Caecum, colon and rectum were obtained and the colon length was measured from the caeco-colonic junction to the anus. After rinsing with PBS the colon was weighed and the weight:length (W:L) ratio (cm/g) was calculated. This was used as an objective parameter indicating the presence of established colitis, as it is known to correlate with histopathology [36]. The proximal 1/3 of the colon was removed and the remaining 2/3 of colon was bisected or trisected longitudinally. The dissected colon biopsies were processed for histological analysis and RNA profiling as previously described [63]. 

### 4.10. Histological Analysis of Tissues

Tissue for histology was fixed in 4% paraformaldehyde (VWR—Bie & Berntsen, Herlev, Denmark) for approximately 24 h at 4 °C. Subsequently, the samples were transferred to 70% ethanol and stored at 4 °C until processed for histopathology. Paraffin embedded tissue blocks were sectioned at a nominal thickness of 3 μm, and mounted on Superfrost^®^Plus microscope slides. Subsequently, the slides were stained with haematoxylin (Ampliqon, Skovlunde, Denmark) and eosin (Sigma-Aldrich, Broendby, Denmark) (H&E) for light-microscopic examination, using an Olympus AX70 microscope. The severity of the histopathological lesions of colon segments was examined in a blinded manner, using the criteria previously described [63].

### 4.11. In Situ Hybridization

Tissues were prepared as described above. Two sets of RNAscope^®^ (Advanced Cell Diagnostics, ACD, Newark, CA, USA) probes were designed to target exon 9 and 10 of either the human or the mouse GHR transcript. Stains were performed on the Ventana Discovery Ultra (Ventana Medical Systems, Inc., Tucson, AZ, USA) with probes targeting bacterial gene *dapB* as negative control. Development was performed using Fast Red. 

### 4.12. Image Analysis and Quantification of Stains

Scanning of ISH stains was done on the Nanozoomer 2.0 (Hamamatsu Photonics K.K., Hamamatsu City, Japan). Using an original magnification of 40× the images were digitally analysed using the software Visiopharm Integrator System (VIS, Visiopharm, Hoersholm, Denmark), as we have described previously [35]. We performed a threshold analysis using an intensity interval of 0–180.

### 4.13. RNA Extraction from Human Biopsies, Murine Intestine and Cell Cultures

Human biopsies and murine intestines were kept in RNAlater (Ambion, Austin, TX, USA) 24–48 h before storage at −80 °C. RNA from human biopsies was extracted by mechanical homogenization using a rotor-stator. RNA purification was performed by column purification (Nucleospin miRNA, Macherey–Nagel, Düren, Germany, ref: 740971.250) including DNAse I treatment according to the recommendations of the manufacturer.

Murine colonic samples for RNAseq were lysed in QIAzol (Qiagen, Valencia, CA, USA) or in RLT lysis buffer added β-mercaptoethanol (BME) (Qiagen, Valencia, CA, USA). Following sample homogenization the samples in QIAzol or RLT plus BME were added chloroform or acid phenol:chloroform, respectively. Following centrifugation the sample supernatant were added 70% ethanol and total mRNA was isolated the Qiagen RNeasy Mini kit protocol (Qiagen, Valencia, CA, USA) including DNase treatment (Ambion, Austin, TX, USA). 

The RNA concentration from human and murine tissues was quantified using a NanoDrop spectrometer (Thermo Fisher Scientific, Wilmington, DE, USA). For samples with <10 ng/mL the Ribogreen^®^ RNA assay Kit (Life Technologies, Carlsbad, CA, USA) was used. The Bioanalyzer (Agilent Technologies, Santa Clara, CA, USA) was used to assess RNA quality. Samples with a resulting RNA Integrity Number (RIN) value below 6 were excluded. RNA from cell cultures was extracted using the GeneJET-kit (Thermo Fisher, K0732) according to the manufacturer’s recommendations.

### 4.14. mRNA Sequencing Analysis and qPCR (Murine)

For preparation of the mRNA sequencing libraries, we sequenced a total of 100 ng RNA per sample using the HiSeq 2000 system (Illumina, San Diego, CA, USA) and the Illumina TruSeq Sample Prep Kit according to the manufacturer’s recommendations. The analyses were run as multiplexing sequencing sufficient to obtain 10–25 million reads per sample. The reference mouse genome (NCBI Build 37, mm9) was used for alignment using the Tuxedo suite [64,65]. Using R (version 3.2.3, available online: http://www.r-project.org), the ShortRead package as well as the Fastx toolkit were used for quality control of the obtained sequencing data [66]. Counts of reads mapping to each gene were calculated from the aligned reads using HTseq [66].

For qPCR on primary murine cell cultures, 200 ng RNA was converted to cDNA using the High-Capacity cDNA Reverse Transcription Kit (4368814, Thermo Fisher). Mastermix (4369514, Thermo Fisher) and Taq-Man probe-primer sets were from Thermo Fisher. The analysis was performed using the (StepOnePlus™ Real-Time PCR system (Applied Biosystems, Foster City, CA, USA). Calculation of expression levels are based in the ΔΔ*C*t method using Rplp0.

### 4.15. mRNA qPCR and Array Analysis (Human)

Quantitative real time PCR (qPCR) of human patient samples were conducted using the Fluidigm Biomark™ System (Fluidigm, South San Francisco, CA, USA) with standard Taq-Man probe-primer sets from Thermo Fisher. Calculation of expression levels are based in the ΔΔ*C*t method using RPLP0.

RNA form human biopsies was further analysed using the Affymetrix (Santa Clara, CA, USA) HGU219 arrays on 96-PEG plates. The average expression level of 2 individual biopsies were calculated and used in the later analysis. 100 ng of RNA was labelled using the 3-IVT expression kit (Affymetrix) and hybridized to the HGU219 chip according to the Affymetrix protocol. The chips were scanned on the GeneTitan platform (Affymetrix).

The Affymetrix raw data (.cel files) were normalized using the robust multichip average (RMA) algorithm [67] in R environment. Quality assurance was performed using the Bioconductor package arrayQualityMetrics in R [68], leading to removal of 2 biopsies prior to data analysis. Probe set annotation files were obtained form from Affymetrix NetAffx website (NA35, available online: https://www.affymetrix.com/analysis/index.affx).

### 4.16. Serum Measurements

TNF-α, IL-1β and IL-6 in patient serum was measured using a plasmonic resonance protein microarray developed at Stanford University [69,70].

### 4.17. Cell Culture Procedures and Western Blotting

Small intestinal enteroid/organoid cultures were established from wild type C57/Bl6 mice and from Ghr-disrupted littermates [71]. The cultures were established and propagated as described previously [48] including mR-spondin (10% *v*/*v*, ~500 ng/mL, homemade), mEGF (50 ng/mL, Peprotech (Rocky Hill, NJ, USA), 315-09), mNoggin (100 ng/mL, Peprotech, 250-38), *N*-Acetylcysteine (1 mM, Sigma-Aldrich, A9165) and 1× B27 supplement without insulin (Gibco (Gaithersburg, MD, USA), A18956-01). Y-27632 (10 µM, Sigma-Aldrich, Y0503) was added for two days after passaging, except when the matrigel was changed prior to stimulation the following day. The cultures were treated with murine forms of TNF-α, IL-1β and IL-6 (1 nM, R&D Systems (Minneapolis, MN, USA), 401-ML, 406-ML, CS1315031-ML); the concentrations of 1 nM used in these experiments are assumedly a reachable local intestinal concentration during inflammation based on ELISA measurements of inflamed rodent intestines [72,73]. Murine GH (200 ng/mL) was produced in-house by Novo Nordisk.

For gene expression analysis, the cells were gently extracted manually from the matrigel 18 h before the experiment and placed in fresh matrigel and media without EGF and R-spondin. The cells were added the inflammatory mediators in basal medium only added *N*-Acetylcysteine for 8 h and RNA was extracted and analysed as described above.

For detection of phosphorylated STAT5 by western blotting, the cells were initially treated as for PCR analysis. After incubation with inflammatory mediators cells were extracted using Cell Recovery Solution (Corning (Tewksbury, MA, USA), 354253) on ice, washed with basal medium and added pre-warmed basal medium containing 200 ng/mL mGH for 40 min before wash and lysis using Cell lysis buffer (Cell signalling technology (Danvers, MA, USA), 9803) added protease and phosphatase inhibitors (Thermo Scientific, 186209) (Cell signalling technology, 5870). The lysate was sonicated and cleared by centrifugation and loaded on homemade 8% SDS-PAGE gels following boiling and reduction. 15 μg of protein was loaded and the following antibodies from Cell Signalling Technologies were used; p-STAT5 (1:1000, 9351), β-Actin (1:2000, 4970) and α-Rb-HRP (1:2000, 7074). Development was performed using SuperSignal™ West Femto Maximum Sensitivity Substrate (Thermo Fisher, 34095) and X-ray film (Bioland (Paramount, CA, USA), A03-01).

### 4.18. Statistical Analyses

GraphPad Prism version 7.0 (GraphPad Software, Inc., La Jolla, CA, USA) was used for all statistical analyses. Non-Gaussian distribution was assumed for the human patient data and the enteroid studies. Therefore, multiple group comparisons were done using non-parametric Kruskal-Wallis with Dunn’s correction for multiple testing. Mann-Whitney *U* tests were used when comparing only two groups. Based on normality testing Gaussian distribution was assumed in data generated form the PAC model, where ordinary one-way ANOVA w. Holm-Sidak’s correction was used when comparing multiple relevant groups. Error bars represent mean ± SD, except SEM is shown in weight change curves for the in vivo studies. Correlation analyses were done using simple linear regression. Significance was assumed with *p*-values < 0.05. 

## 5. Conclusions

Expression analyses of human UC patients and the PAC experimental colitis model revealed similarities among deregulated transcripts involved in GH signalling and the formation of GH resistance. Resistance appears to be a result of increased SOCS1 and SOCS3 expression and down-regulated GHR expression, mediated by the inflammatory mediators TNF-α, IL-1β and IL-6. These results were confirmed in cultured primary epithelial cells. Consequently, inflammation likely has a direct impact on mucosal healing during intestinal inflammation by impairing local GH action. Therapeutic treatment of murine colitis using mGH and PEG-hGH did increase body weight, but no effects were observed on mucosal healing. Accordingly, GH does not reduce intestinal inflammation in our mouse models, potentially due to established GH resistance. Treatment of the underlying inflammation may therefore be important in order to sensitize the intestinal cells to regain responsiveness to GH.

## Figures and Tables

**Table 1:** Demographic data and disease characteristics of ulcerative colitis (UC) patients and controls.

Clinical Variables	Controls	Remission	Mild	Moderate	Severe	*p*-Value
	*n* = 20	*n* = 21	*n* = 11	*n* = 24	*n* = 10	
Gender (male/female)	7/13	12/9	4/7	11/13	3/7	=0.52 °
Age, years (median, IQR)	49 (20)	52 (27)	31 (15)	40 (22)	30 (14)	<0.01 ^Δ^
Mayo score (median, IQR)	0 (0)	0 (1)	4 (2)	8 (3)	12 (1)	<0.001 ^Δ^
Mayo endoscopic score (median, IQR)	0 (0)	0 (0)	1 (0)	2 (0)	3 (0)	<0.001 ^Δ^
Geboes score (mean, IQR) ^§^	0.4 (1)	1.0 (1)	5.3 (14)	13.0 (10)	19.0 (4.5)	<0.001 ^Δ^
Smoking/non-smoking	4/16	3/18	0/11	0/24	4/6	<0.05 °
Daily medication:						<0.05 °
- Steroids (oral or topical)	0	0	2	10	4	
- 5-ASA (oral or topical)	0	19	7	22	8	
- Thiopurines	0	4	2	8	0	
- Infliximab	0	3	0	1	2	
- Antibiotics	0	0	1	2	3	
- None	40	1	2	0	2	

The patients were divided based on the Mayo disease score into remission (0–2, no subscore > 1), mild (3–5), moderate (6–10) or severe (11–12). IQR: interquartile range; ° Chi-square test; All groups included except controls group when comparing daily medication. ^Δ^ Ordinary one-way analysis of variance (ANOVA) with Dunn’s correction; all groups included. ^§^ The histopathological Geboes score is used as a linear accumulative score ranging from 0 to 22; 5-ASA: 5-aminosalicylic acid.

**Table 2:** Comparison of transcripts across model systems.

Gene	Human (Array) *n* = 19 (H), 32 (I)		Enteroids (qPCR) *n* = 8 (H), 8 (I)		PAC (mRNA Seq) *n* = 3 (H), 12 (I)	
	Mean (SD)	*P*-Value	Mean (SD)	*p*-Value	Mean (SD)	*p*-Value
GHR	H: 55.27 (30.21) I *: 13.94 (4.70)	↓ <0.0001	H: 3.31 (0.47) I °: 1.54 (0.36)	↓ <0.001	H: 10.06 (0.39) I ^Δ^: 5.37 (1.40)	↓ <0.01
PRLR	H: 20.18 (4.77) I *: 10.69 (2.51)	↓ <0.0001	H: 11.21 (2.24) I °: 5.50 (1.46)	↓ <0.001	H: 1.45 (0.13) I ^Δ^: 0.28 (0.23)	↓ <0.01
TNF-α	H: 25.63 (7.07) I *: 49.19 (31.74)	↑ <0.0001	-		H: 0.57 (0.15) I ^Δ^: 12.60 (9.03)	↑ <0.01
IL-1β	H: 48.20 (49.94) I *: 643.1 (952.2)	↑ <0.0001	-		H: 1.37 (0.32) I ^Δ^: 38.04 (40.41)	↑ <0.01
IL-6	H: 8.32 (4.33) I *: 29.41 (81.64)	↑ <0.0001	-		H: 0.027 (0.046) I ^Δ^: 1.014 (1.008)	↑ <0.05
SOCS1	H: 8.86 (2.86) I *: 35.18 (18.08)	↑ <0.0001	H: 2.43 (0.24) I °: 3.21 (0.25)	↑ <0.001	H: 2.93 (0.84) I °: 51.76 (32.83)	↑ <0.01
SOCS2	H: 64.65 (14.17) I *: 43.42 (11.96)	↓ <0.0001	-		H: 9.56 (0.91) I ^Δ^: 4.02 (0.87)	↓ <0.01
SOCS3	H: 4.89 (1.17) I *: 9.90 (5.66)	↑ <0.0001	H: 6.14 (0.72) I °: 7.47 (0.80)	↑ <0.01	H: 6.50 (2.01) I ^Δ^: 76.34 (36.57)	↑ <0.01
IGF-1	H: 32.23 (7.57) I *: 32.21 (10.49)	=0.9 (ns)	H: Below detection limitI °: Below detection limit		H: 2.20 (0.72) I ^Δ^: 3.05 (1.20)	=0.3 (ns)
IGF1R	H: 28.25 (4.18) I *: 23.01 (4.53)	↓ <0.0001	H: 1.18 (0.17) I °: 1.56 (0.32)	↑ <0.05	H: 3.34 (0.22) I ^Δ^: 2.34 (0.31)	↓ <0.01
GH1	Below detection limit (pcr)		-		Below detection limit	
GH2	H: 8.35 (1.34) I *: 7.91 (0.92)	=0.5 (ns)	H: 0.02 (0.02) I °: 0.02 (0.02)	=0.9 (ns)	ND ^§^	

Comparison of gene expression profiles of selected transcript across the three model systems; human ulcerative colitis, mouse enteroids and murine PAC (colitis model). H: “healthy” samples with no inflammation. I: Samples with inflammation present. Statistics are Mann–Whitney *U*-test, two-tailed. * Comparing non-IBD controls with patients having moderate-severe colitis. ° Comparing non-treated enteroids with enteroids treated with TNF-α, IL-1β and IL-6. ^Δ^ Comparing C57BL/6j controls with PAC animals at day 10. ↓ means down-regulated, ↑ means up-regulated upon inflammation. ^§^ Not available from sequencing data.

## Supplementary Materials

Supplementary materials can be found at www.mdpi.com/1422-0067/18/10/2046/s1.
