# Supplementary material for: Characterization of Growth Hormone Resistance in Experimental and Ulcerative Colitis
Source: Int J Mol Sci. 2017 Sep 23;18(10):2046. doi: 10.3390/ijms18102046 (PMC5666728; doi:10.3390/ijms18102046)
Supplement: Supplementary file 1 [file ijms-18-02046-s001.pdf]

Supplementary Materials

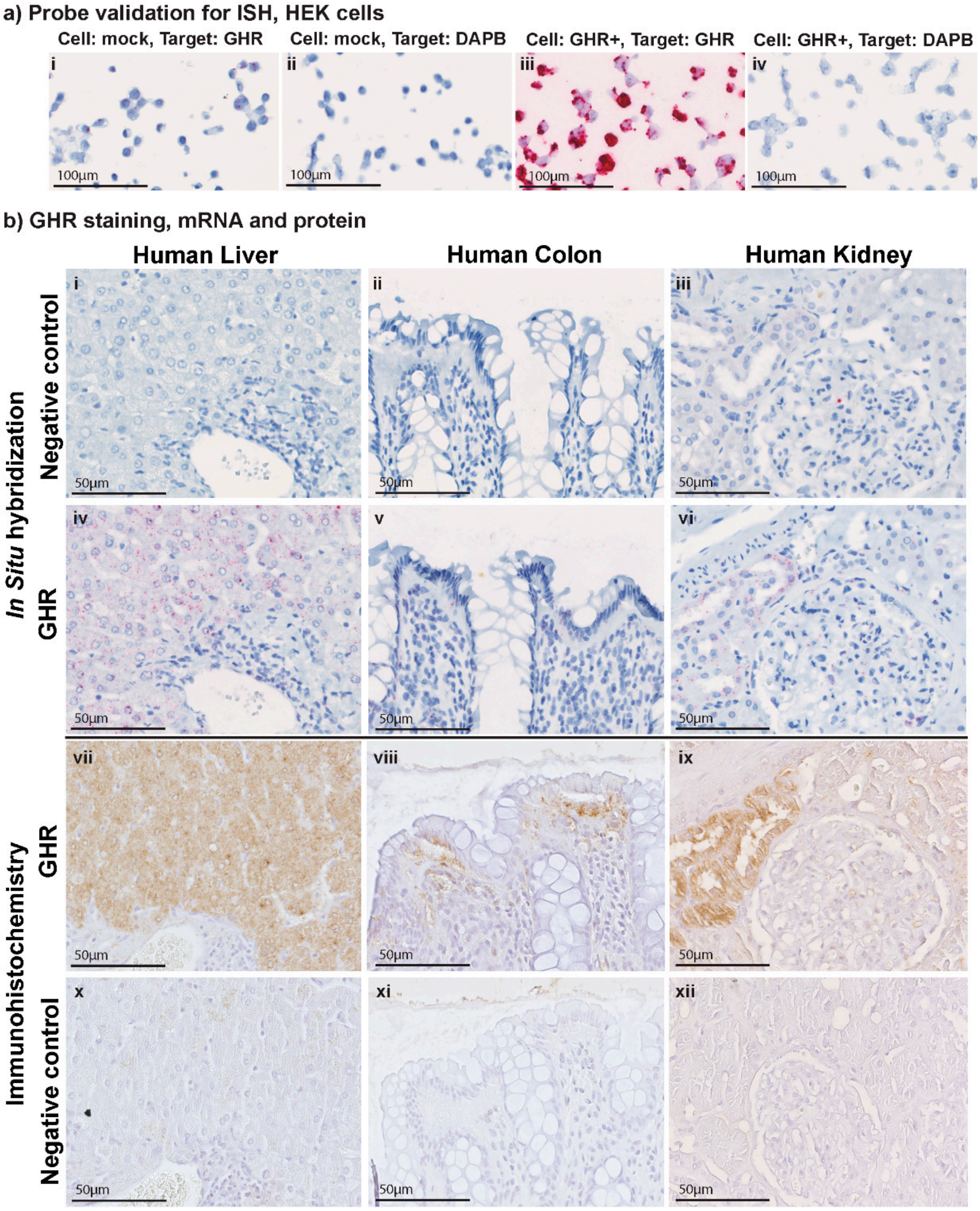

Figure S1. Validation of ISH and IHC for detection of GHR transcript and protein. (**a**) Validation of ISH probes using mock-transfected (**i**, **ii**) and GHR transfected (**iii**, **iv**) HEK cells. HEK cells express endogenous GHR and are not expected to be negative for GHR transcript. Probe sets targeting hGHR (**i**, **iii**) and bacterial DAPB (**ii**, **iv**) as negative control; (**b**) Staining of human liver (**i, iv, vii, x**), colon (**ii, v, viii, xi**) and kidney (**iii, vi, ix, xii**) by ISH (top panel (**i-vi**)) and IHC (bottom panel (**vii-xii**) using the ISH probes validated in a) and the monoclonal antibody MAB263, respectively. IHC protocol was adapted from "J Clin Endocrinol Metab. 1995 Nov; 80(11):3361-7" including vestastain-HRP (PK-6100, Vector Labs Burlingame, CA, USA) and TSA amplification (NEL700001KT, Perkin Elmer, Skovlunde, Denmark). The two methods of staining (ISH and IHC) show overlapping patterns.

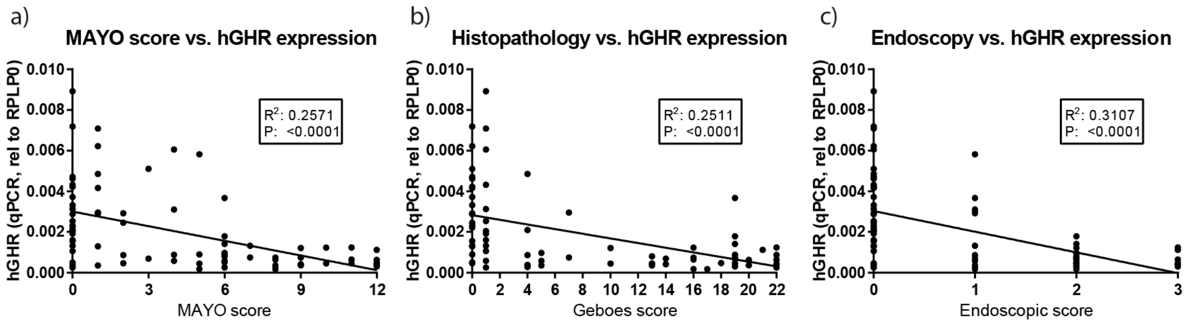

Figure S2. Correlation between hGHR expression and disease severity. Correlation between hGHR expression (qPCR data) and three measures of disease severity; global MAYO score, histopathological evaluation using the Geboes system and endoscopy. GHR expression correlates inversely with all of them.

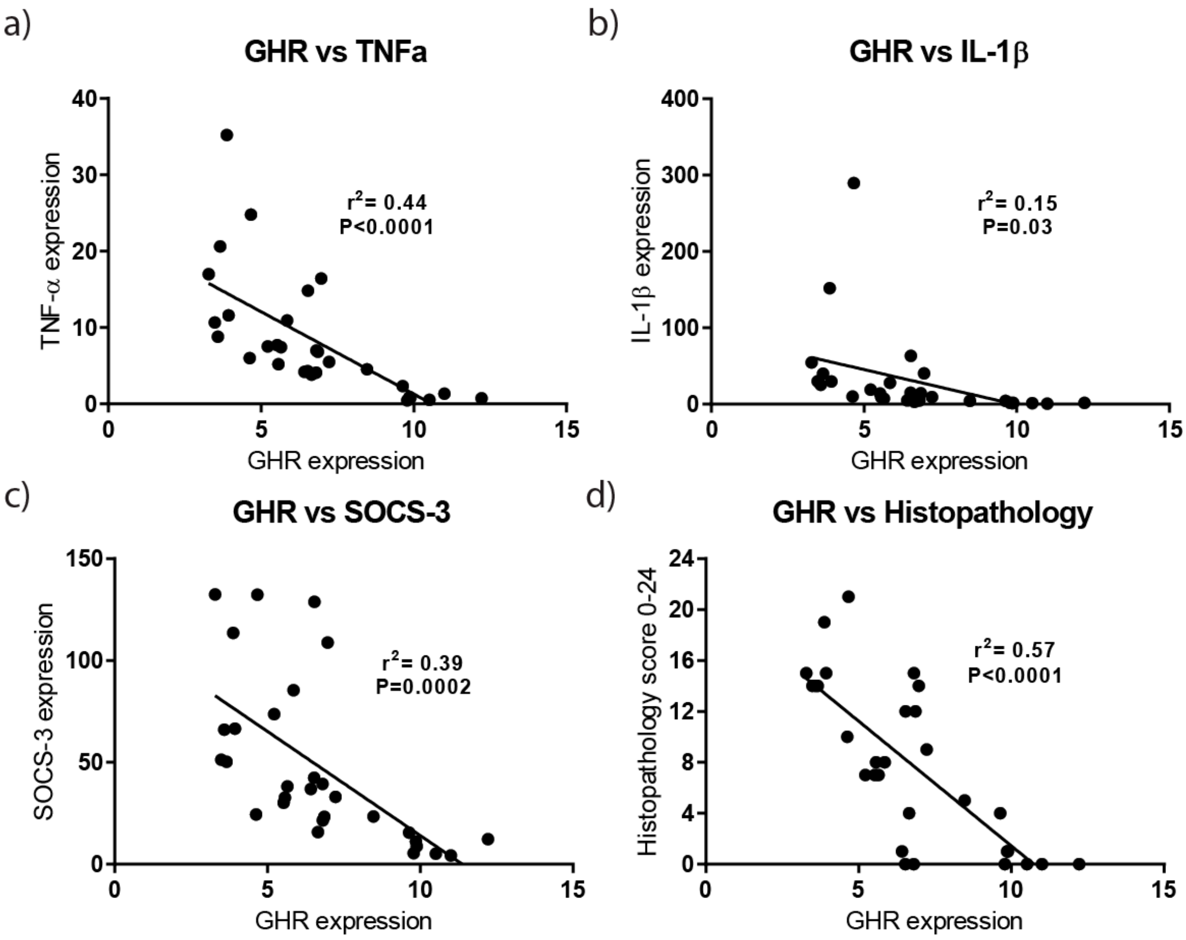

Figure S3. mGHR expression versus relevant gene expression and histopathology. Colonic expression of mGHR from the murine PAC model, derived from sequencing data. Correlations are shown to the local expression of TNF-α (**a**), IL-1β (**b**) and SOCS3 (**c**) and to the histopathological disease score (**d**). All four measures correlate inversely with mGHR expression.

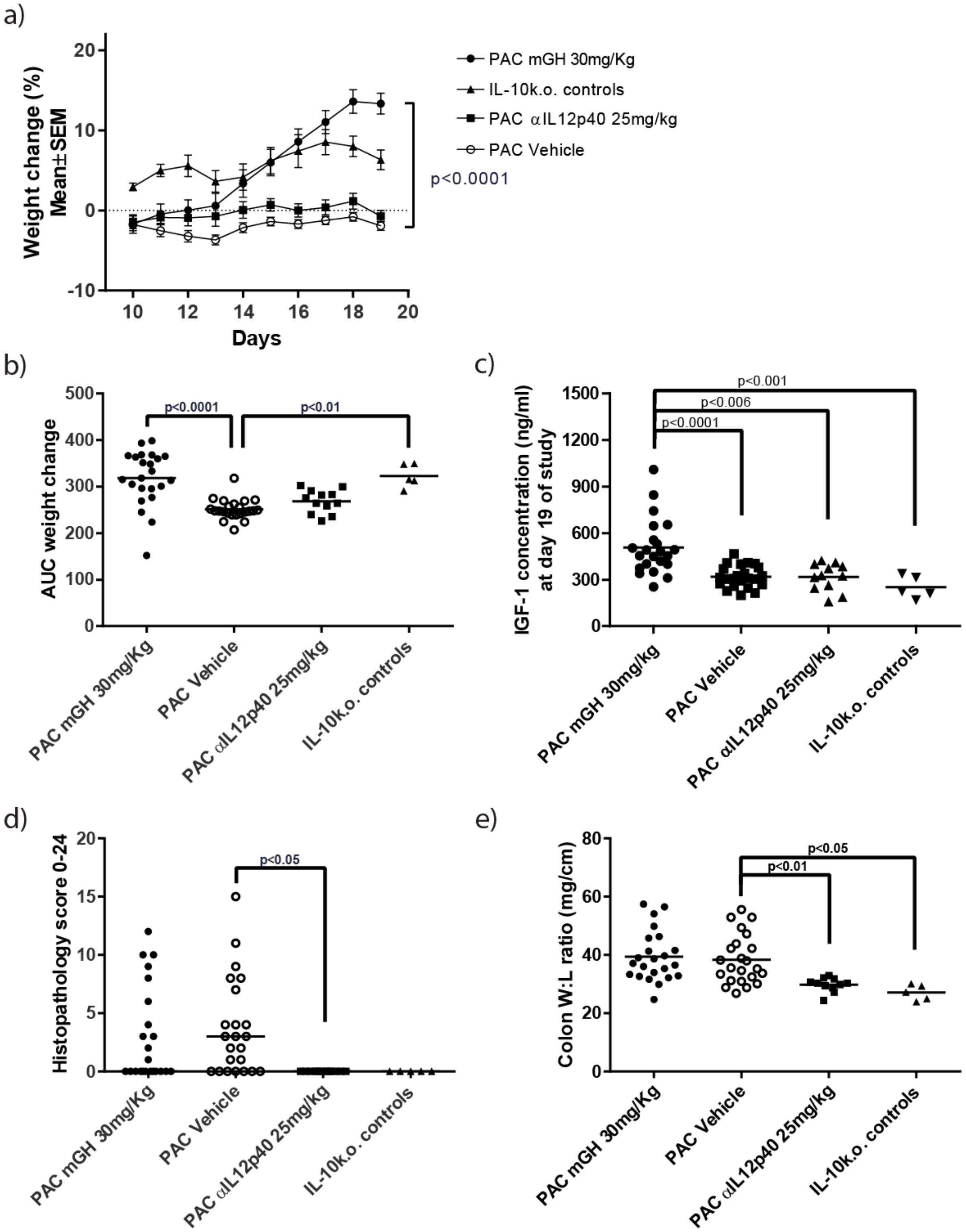

Figure S4. Therapeutic evaluation of the effects of twice daily mGH in the PAC model with established colitis. (**a**) Weight change (% relative to pre-dosing with piroxicam) during the study. The four treatment groups are indicated; (**b**) Area under the curve (AUC) showing the body weight for the animals in the treatment groups during the study; (**c**) Plasma IGF-1 at day 19 (following 9 consecutive days of mGH treatment); (**d**) Histopathological scoring of colonic disease severity evaluated following 10 days of mGH treatment; (**e**) Colonic weight:length ratio of the treated groups.
